# DYRK1A inhibition results in MYC and ERK activation rendering *KMT2A-*R acute lymphoblastic leukemia cells sensitive to BCL2 inhibition

**DOI:** 10.1038/s41375-025-02575-w

**Published:** 2025-03-27

**Authors:** V. S. S. Abhinav Ayyadevara, Gerald Wertheim, Shikha Gaur, John A. Chukinas, Joseph P. Loftus, Sung June Lee, Anil Kumar, Srividya Swaminathan, Rahul S. Bhansali, Wayne Childers, Huimin Geng, Thomas A. Milne, Xianxin Hua, Kathrin M. Bernt, Thierry Besson, Junwei Shi, John D. Crispino, Martin Carroll, Sarah K. Tasian, Christian Hurtz

**Affiliations:** 1 Department of Basic Science, Division of Cancer Sciences, Loma Linda University School of Medicine, Loma Linda, CA USA; 2 Department of Pathology and Laboratory Medicine, University of Pennsylvania Perelman School of Medicine, Philadelphia, PA USA; 3 Division of Oncology and Center for Childhood Cancer Research, Children’s Hospital of Philadelphia, Philadelphia, PA USA; 4 Department of Systems Biology, City of Hope Beckman Research Institute, Duarte, CA USA; 5 Department of Pediatrics, City of Hope Beckman Research Institute, Duarte, CA USA; 6 Department of Medicine, University of Pennsylvania Perelman School of Medicine, Philadelphia, PA USA; 7 Moulder Center for Drug Discovery, Temple University School of Pharmacy, Philadelphia, PA USA; 8 Department of Laboratory Medicine, University of California, San Francisco, CA USA; 9 MRC Molecular Haematology Unit, MRC Weatherall Institute of Molecular Medicine, Radcliffe Department of Medicine, University of Oxford, Oxford, UK; 10 Department of Cancer Biology, Abramson Family Cancer Research Institute, Perelman School of Medicine at the University of Pennsylvania, Philadelphia, PA USA; 11 Department of Pediatrics and Abramson Cancer Center at the Perelman School of Medicine at the University of Pennsylvania, Philadelphia, PA USA; 12 Univ Rouen Normandie, INSA Rouen Normandie, CNRS, Institut CARMeN UMR 6064, Rouen, France; 13 Division of Experimental Hematology, St Jude Children’s Research Hospital, Memphis, TN USA; 14 Princess Máxima Center for Pediatric Oncology, Utrecht, the Netherlands

**Keywords:** Targeted therapies, Cell signalling

## Abstract

Unbiased kinome-wide CRISPR screening identified DYRK1A as a potential therapeutic target in *KMT2A*-rearranged (*KMT2A-*R) B-acute lymphoblastic leukemia (ALL). Mechanistically, we demonstrate that *DYRK1A* is regulated by the KMT2A fusion protein and affects cell proliferation by regulating MYC expression and ERK phosphorylation. We further observed that pharmacologic DYRK1A inhibition markedly reduced human *KMT2A*-R ALL cell proliferation in vitro and potently decreased leukemia proliferation in vivo in drug-treated patient-derived xenograft mouse models. DYRK1A inhibition induced expression of the proapoptotic factor BIM and reduced the expression of BCL-XL, consequently sensitizing *KMT2A*-R ALL cells to BCL2 inhibition. Dual inhibition of DYRK1A and BCL2 synergistically decreased *KMT2A*-R ALL cell survival in vitro and reduced leukemic burden in mice. Taken together, our data establishes DYRK1A as a novel therapeutic target in *KMT2A*-R ALL and credential dual inhibition of DYRK1A and BCL2 as an effective translational therapeutic strategy for this high-risk ALL subtype.

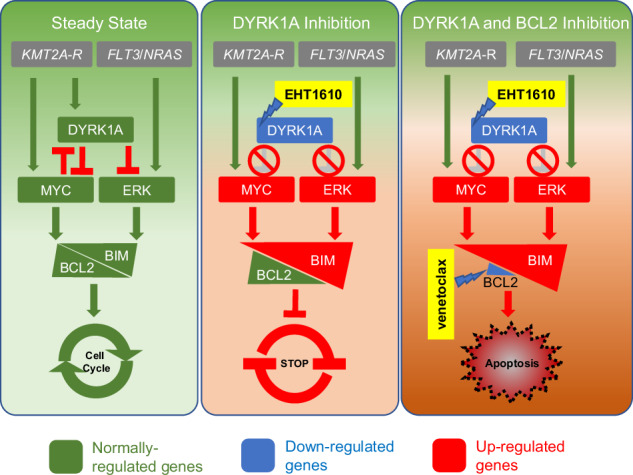

## Introduction

Rearrangements in *KMT2A* (formerly *MLL*) occur in ~75% of acute lymphoblastic leukemia (ALL) cases in infants less than 12 months of age, 1–2% of older children, and 10% of adults [1–3]. *KMT2A*-rearranged (*KMT2A-*R) ALL is a high-risk disease associated with frequent chemoresistance and poor clinical outcomes in most patients with a survival rate of <75% in children and <35% in infants and adults. We thus focused in these studies on identifying new biological drivers and therapeutic vulnerabilities in this high-risk ALL subtype.

The use of small molecule inhibitors targeting activated and disease-driving kinases has demonstrated significant clinical success in lymphoid malignancies. For instance, targeting the *BCR::ABL1* oncogene in patients with Philadelphia chromosome-positive (Ph+) ALL has significantly improved their event-free and overall survival [4, 5]. Similarly, BTK inhibition with ibrutinib significantly improved the survival of patients with chronic lymphocytic leukemia [6]. Therapeutic targeting of FLT3 and other non-kinase dependencies in *KMT2A*-R ALL, including DOT1L and menin, have demonstrated robust activity in preclinical studies and have been translated to the clinic [7–11]. However, incomplete therapeutic activity and/or failure to improve clinical outcomes have been observed with several of these drugs [12–14]. The rapid development of resistance mutations in inhibitor-treated patients despite initial response has also been reported [15], highlighting a persistent scientific knowledge gap and unmet medical need for better therapies.

Given the success of various small molecule inhibitors in specific genetic subtypes of ALL and acute myeloid leukemia (AML) and the improved specificity of next-generation kinase inhibitors [16–20], we performed a kinome-wide domain-specific CRISPR screen in 3 high-risk subtypes of ALL and found that dual-specificity tyrosine phosphorylation-regulated kinase 1A (DYRK1A) is specifically required for the survival of *KMT2A-*R ALL. DYRK1A is a dual-specificity kinase that autophosphorylates a tyrosine residue for activation [21], but otherwise primarily targets serine/threonine residues on substrates for phosphorylation. The protein has a proposed, but still incompletely defined, role in cell cycle regulation that may vary depending on cell type. Significant research has characterized the role of DYRK1A in neuronal development, and a smaller body of work has also implicated its role in normal B cell differentiation and in Down syndrome (DS)-associated ALL and *CRLF2*-R Ph-like ALL [22–25].

Herein, we demonstrate an unanticipated direct regulation of DYRK1A via oncogenic *KMT2A* rearrangements in ALL and a specific requirement of DYRK1A for leukemia proliferation. We also report that DYRK1A is required to suppress the proapoptotic molecule BIM and that DYRK1A inhibition consequently renders *KMT2A*-R ALL cells sensitive to BCL2 inhibition. Overall, these data offer new insights into the mechanisms underlying *KMT2A* fusion-driven leukemogenesis and may have translational therapeutic implications. This is particularly relevant given that early-stage DYRK1A inhibitors are currently under clinical investigation for osteoarthritis (NCT05603754) and atopic dermatitis (NCT05382819) [26, 27].

## Materials and methods

### Primary human ALL specimens and cell lines

Diagnostic bone marrow specimens from children and adults with ALL were obtained via informed consent on Institutional Review Board-approved research protocols of the Children’s Hospital of Philadelphia (CHOP) and University of Pennsylvania (UPenn) in accordance with the Declaration of Helsinki (Table S1**)** as previously described [28, 29]. Human *KMT2A*-R and non-*KMT2A*-R ALL cell lines (Table S2) were obtained via collaborators or purchased from the DSMZ biorepository (Braunschweig, Germany), validated by short tandem repeat analysis, and confirmed as *Mycoplasma*-free every 6 months [30–33].

Human ALL cells harvested from murine spleens of some PDX models were cultured on OP9 stroma in Minimum Essential Medium (MEMα; Life Technologies) with GlutaMAX containing 20% FBS, 100 IU/mL penicillin, 100 μg/mL streptomycin, and 1 mM sodium pyruvate. Cell lines were cultured in Roswell Park Memorial Institute medium ([RPMI] Life Technologies; Carlsbad, CA) with GlutaMAX containing 10 or 20% FBS, 100 IU/mL penicillin, 100 μg/mL streptomycin (hereafter referred to as ‘B cell medium’) at 37 °C in a humidified incubator with 5% CO_2_ and maintained in culture for fewer than 3 months to minimize infectious contamination and genetic drift.

### Retroviral and lentiviral transduction

Transfections of 293FT cells with retroviral and lentiviral constructs were performed using lipofectamine 2000 with Opti-MEM media (Invitrogen; Carlsbad, CA). Viral supernatants were produced by co-transfecting 293FT cells with the viral gag-pol and packaging vectors together with the Cas9 or sgRNA constructs (Table S3). Cultivation was performed in high glucose Dulbecco’s modified Eagle’s medium ([DMEM] Invitrogen) with GlutaMAX containing 10% fetal bovine serum, 100 IU/mL penicillin, 100 μg/mL streptomycin, 25 mM HEPES, 1 mM sodium pyruvate, and 0.1 mM non-essential amino acids. Regular media were replaced after 16 h by growth media containing 3 mM caffeine. After 24 h, the viral supernatant was harvested and filtered through a 0.45 μm filter. Retroviral transductions were performed by loading the viral supernatant on 50 μg/mL retronectin-coated (Takara) non-tissue culture-treated 6-well plates prior to centrifugation (2000 × *g*, 90 min at 32 °C) two times. Subsequently, 1–2 × 10^6^ cells were loaded and transduced per well by centrifugation at 600 × *g* for 30 min and maintained for 72 h at 37 °C with 5% CO_2_ prior to transfer into culture flasks. Lentiviral transductions were performed by loading viral supernatant on 50 μg/mL retronectin-coated non-tissue culture-treated 6-well plates with 1–2 × 10^6^ cells. Cells were centrifuged ×30 min at 600 × *g* and maintained for 16 h at 37 °C with 5% CO_2_ prior to replacing media with fresh RPMI and GlutaMAX containing 20% FBS, 100 IU/mL penicillin, and 100 μg/mL streptomycin. Cells were then cultured at 37 °C with 5% CO_2_ prior to analysis.

## Results

### KMT2A-R ALL is dependent on DYRK1A expression for survival

**Fig. 1:**
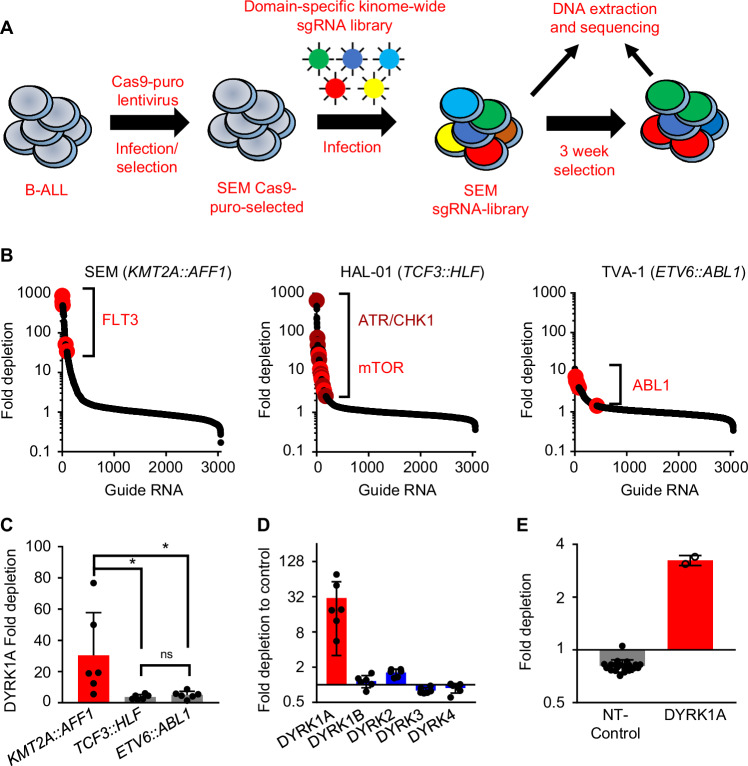
Domain-specific kinome-wide CRISPR screen in high-risk ALL subtypes. **A** Overview of the performed domain-specific kinome-wide library screen. SEM (*KMT2A::AFF1*), HAL-01 (*TCF3::HLF*), and TVA-1 (*ETV6::ABL1*) cells were virally transduced with a lentiviral Cas9-Puro construct. After puromycin selection, the cells were transduced with a GFP-tagged domain-specific kinome-wide CRISPR library consisting of 550 kinases, 100 negative controls, and 50 positive controls (6 guide RNAs (sgRNA) per kinase). Cells were collected at 2 time points for sequencing; 3 days after the library transduction, which served as input control, and 3 weeks after the transduction which served as the experimental readout. **B** Shown is the fold depletion of the individual sgRNAs in the tested samples. Highlighted are known positive controls for each individual ALL subtype. **C** Shown is the fold depletion of *DYRK1A* among the different ALL subtypes. **D** Shown is the fold depletion of each *DYRK* family member in SEM cells. **E** Cas9-puro-selected SEM cells were lentivirally transduced with a smaller sgRNA-GFP library. The library included two sgRNAs targeting one of 14 previously identified targets including 21 negative controls. Data are represented as individual values with mean ± SEM bars. *P < 0.05; **P < 0.01; ***P < 0.001 by *t*-test.

We performed a domain-specific kinome-wide CRISPR screen in genetically diverse high-risk ALL cell lines SEM (*KMT2A::AFF1*) and HAL-01 (*TCF3::HLF*) and TVA-1 (*ETV6*::*ABL1*) cells previously immortalized from a Ph-like ALL patient-derived xenograft (PDX) model [30] to identify novel druggable targets in high-risk ALL (Fig. 1A). As expected, we identified dependencies upon (1) FLT3 in the *FLT3* wild-type overexpressing SEM cell line, (2) the checkpoint inhibitors *ATR* and *CHK1* in the HAL-01 ALL cell line, and (3) *ABL1* in TVA-1 cells, validating the specificity of our screens (Fig. 1B and Supplementary Fig. 1A). Unexpectedly, sgRNAs targeting *DYRK1A* were reduced in SEM cells compared to the other tested samples (Fig. 1C**)**. *DYRK1A* is not a common essential gene validated via the Cancer Dependency MAP website, indicating that targeting DYRK1A may represent a target with a unique therapeutic window (Supplementary Fig. 1B). Five different *DYRK* family members are known, but only *DYRK1A* was required for *KMT2A-AFF1* cell survival (Fig. 1D). The importance of *DYRK1A* was validated in a second CRISPR screen consisting of 14 kinases that we identified in our initial screen (Fig. 1E). Taken together, our screening results demonstrate a unique dependency of *KMT2A::AFF1* cells upon *DYRK1A*.

### KMT2A directly binds to the DYRK1A promoter and regulates its expression

**Fig. 2:**
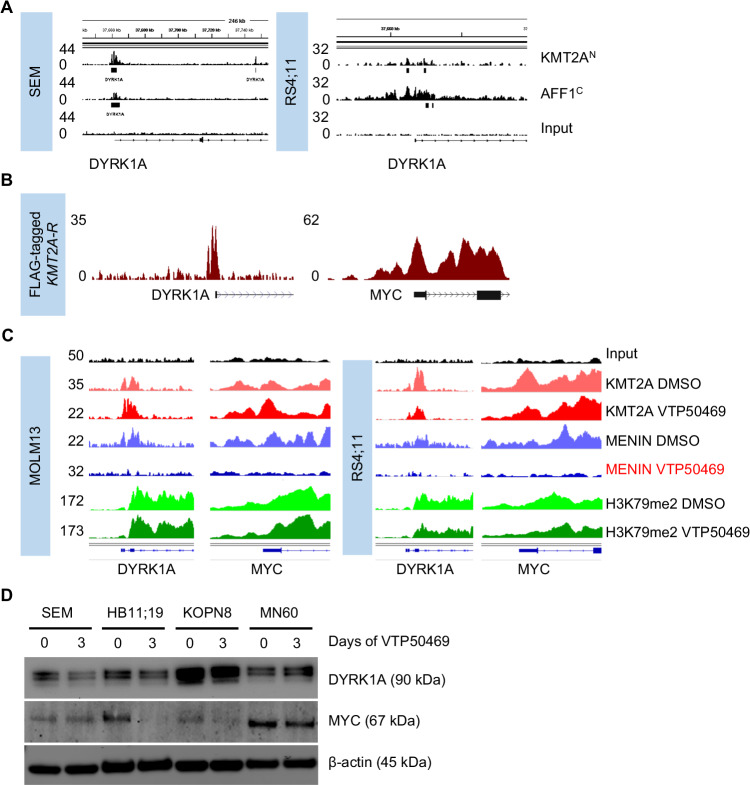
Oncogenic *KMT2A* rearrangements transcriptionally regulate DYRK1A in ALL. **A** ChIP-Seq (Chromatin immunoprecipitation [ChIP] combined with high-throughput sequencing) tracks on the DYRK1A promoter region using antibodies specific for the *KMT2A* N- and the AFF1 C-terminal domain(s) were used. The Y-axis represents the number of reads for peak summit normalized by the total number of reads per track. Data from two *KMT2A*-AFF1 cell lines SEM (GSE83671) [34] and RS4;11 (GSE38403) [35] are shown. **B** ChIP-Seq tracks on the *DYRK1A* and *MYC* promoter regions using a FLAG-specific antibody in *KMT2A*-*Aff1*-FLAG-transformed human ALL cells. The Y-axis represents the number of reads for peak summit normalized by the total number of reads per track (GSE84116) [36]. **C** ChIP-Seq tracks on the *DYRK1A* and *MYC* promoter regions using *KMT2A* N-terminal-, menin-, and H3K79me2-specific antibodies. Two *KMT2A*-R cell lines (MOLM13/AML and RS4;11/ALL) were either treated with control or the Menin inhibitor VTP50469 as previously described (GSE127508) [10]. **D** Western blot analysis of DYRK1A, MYC, and β-actin in three *KMT2A*-R ALL cell lines (SEM, KOPN8, RS4;11) and the IgG-MYC rearranged ALL cell line MN60. Each cell line was treated with either control or the menin inhibitor VTP50469 (100 nM) for 72 h.

Given this specific identification of DYRK1A as a potential driver in *KMT2A*-R ALL via our CRISPR screen, we then hypothesized that oncogenic *KMT2A* rearrangements may directly regulate DYRK1A expression. Concordant with our hypothesis, analysis of previously published ChIP-Seq data of *KMT2A::AFF1* ALL cells demonstrated that both *KMT2A* and *AFF1* directly bind to the promoter region of *DYRK1A* in ALL cell lines SEM and RS4;11 (Fig. 2A) [34, 35]. For the ChIP-Seq experiment, antibodies binding to the N-terminal region of *KMT2A* and the C-terminal region of *AFF1* were used. Given that *KMT2A*-R ALL is often a monoallelic balanced translocation with wild-type *KMT2A* and *AFF1* still present in the cells, we then analyzed a ChIP-Seq data set that used human *KMT2A-*R leukemia cells generated via overexpression of a *KMT2A-Aff1*-flag construct [36]. Using a FLAG antibody, we identified oncogene-specific binding partners and confirmed that *KMT2A-Aff1* directly binds to the *DYRK1A* promoter region, validating that KMT2A fusions can interact directly with the *DYRK1A* promoter (Fig. 2B). The binding of the KMT2A fusion construct to *MYC* is shown as a control.

To validate that KMT2A fusions not only bind to the promoter region but also directly affect DYRK1A transcription, we next analyzed ChIP-Seq data from the MOLM13 (*KMT2A::MLLT3*) AML cell line and the RS4;11 (*KMT2A::AFF1*) ALL cell line [10]. Cells were treated with vehicle or the menin inhibitor VTP50469 (a prolog of the clinical drug revumenib) [10] for 3 days. As expected, menin inhibition prevented the binding of menin, an important regulatory unit that is required for the transcriptional regulation of KMT2A fusion’s target genes, to the *DYRK1A* promoter. *MYC* was analyzed as a positive control demonstrating reduced binding of menin to the *MYC* promoter site and H3K79me2 was analyzed demonstrating the binding of DYRK1A is associated with an active promoter region (Fig. 2C). Of note, other DYRK family members are not regulated via *KMT2A*-R with the exception of *DYRK2* where minimal binding activity was observed (Supplementary Fig. 2C). We next analyzed gene expression data from SEM cells treated with either the DOT1L inhibitor EPZ004777, the menin inhibitor MI-2-2, or a combination of both drugs. Our analysis shows that DYRK1A was specifically downregulated following menin inhibition, while DOT1L inhibition only marginally reduced *DYRK1A* expression levels (Supplementary Fig. 1C). We concluded that *KMT2A* fusions transcriptionally regulate *DYRK1A* via menin, but not epigenetically via *DOT1L*.

To validate this finding, we first performed RT-PCR analysis of control and VTP50469-treated SEM cells, which showed that in vitro menin inhibition led to a significant reduction in *DYRK1A* expression and other known transcriptional targets (Supplementary Fig. 1D). We then used CRISPR-mediated deletion of *KMT2A* in both *KMT2A-*R and wild-type (WT) KMT2A ALL cell lines. Our results clearly demonstrate that deletion of both *KMT2A-*R and *KMT2A-*WT decreased *DYRK1A* expression levels, indicating that both *KMT2A*-R *and KMT2A*-WT regulate *DYRK1A* (Supplementary Fig. 1E).

### DYRK1A is highly expressed in KMT2A-R ALL and high expression levels are retained during relapse

We next tested the DYRK1A protein levels across different subtypes of ALL and AML and, interestingly, observed increased DYRK1A expression in *KMT2A*-R ALL compared to the tested *KMT2A* wild-type leukemia cell lines and PDX cases (Supplementary Fig. 1F, G). We then confirmed our cell line findings in cryopreserved cells from established infant and childhood *KMT2A*-R ALL PDX models created from diagnosis (black) and relapse (red) biospecimens and compared them to non-*KMT2A*-R ALL [37]. These data demonstrate that the high DYRK1A protein levels are retained at relapse and are overall elevated in *KMT2A*-R ALL compared to Ph-like ALL (Supplementary Fig. 1H). Finally, we performed a Western blot analysis of *KMT2A*-R ALL cell lines treated with control or 72 h of VTP50469 and observed a reduction in DYRK1A protein levels over time (Fig. 2D). However, menin inhibition did not fully abrogate the DYRK1A protein expression (Fig. 2D) and only affected cell proliferation of *KMT2A*-R ALL cells after multiple days of VTP50469 exposure (Supplementary Fig. 2A). In addition, apoptosis was induced only when high concentrations of VTP50469 were used (Supplementary Fig. 2B), consistent with previous reports [10]. Overall, these results suggest that *KMT2A* fusions in ALL regulate DYRK1A RNA and protein expression levels via direct transcriptional regulation that requires menin binding.

### DYRK1A and MYC negatively regulate each other

**Fig. 3:**
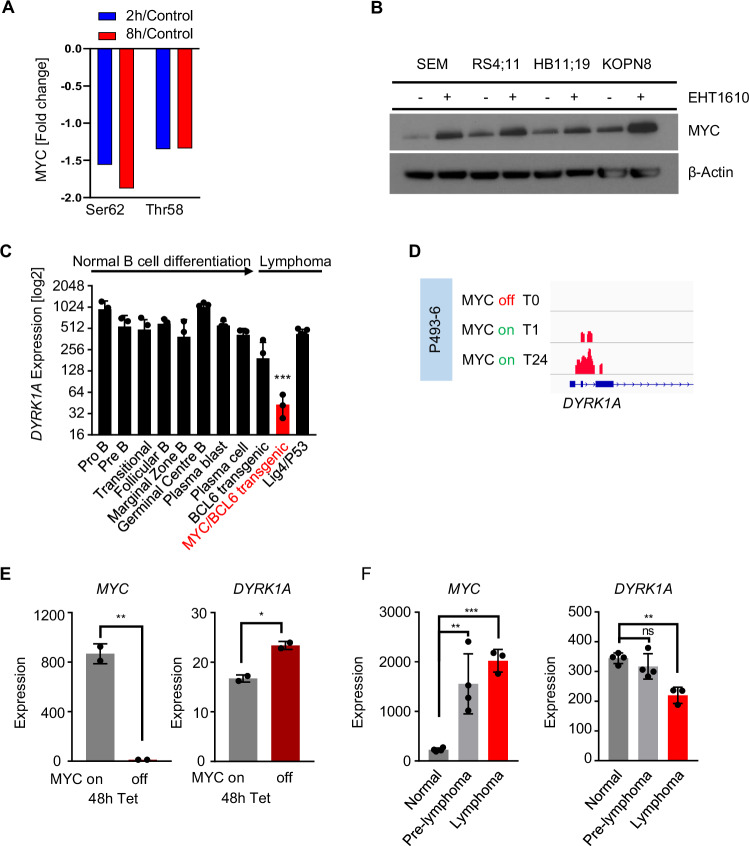
Feedback regulation between DYRK1A and MYC in *KMT2A*-R ALL. **A** Phophoproteomics analysis in HB11;19 cells treated with 5 µM GNF2133 for 2 h and 8 h. **B** Western blot analysis of MYC and β-actin in *KMT2A*-R ALL cell lines treated either with control or EHT1610 (5 µM/72 h). **C** *Dyrk1a* gene expression levels in a comprehensive panel of purified developmentally defined normal murine B cells and genetically distinct murine lymphoma models (GSE26408) [40]. **D** Chip-Seq experiment on the MYC-inducible B cell malignancy model cell line P493-6. Gene tracks of *Myc* binding at the *Dyrk1a* promoter region at 0 h (top), 1 h (middle), and 24 h (bottom) are shown (GSE36354) [43]. **E** *Myc* and *Dyrk1a* gene expression levels in P493-6 cells before and after MYC inactivation. *Myc* inactivation was induced via tetracycline treatment for 48 h (GSE120246). **F** *Myc* and *Dyrk1a* gene expression levels in Eμ-*myc* transgenic mice. Data are represented as individual values with mean ± SEM bars. *P < 0.05; **P < 0.01; ***P < 0.001 by *t*-test.

Viral overexpression of DYRK1A has also been shown to mediate MYC phosphorylation (Ser62 and Thr58) and consequent MYC degradation in non-*KMT2A*-R AML [38], suggesting that DYRK1A may negatively regulate MYC also in ALL. To validate further the observed DYRK1A-mediated regulation of MYC, we used an orthogonal approach by inhibiting DYRK1A and conducting a phosphoproteomics study. This analysis showed reduced phosphorylation at Ser62 and Thr58 at both two hours and eight hours compared to control cells (Fig. 3A). To confirm this finding, we performed a Western blot analysis to assess MYC expression levels following treatment with the DYRK1A inhibitor EHT1610 (without modifying KMT2A fusion activity [39]). As expected, this treatment led to significant MYC upregulation/accumulation across all tested *KMT2A*-R ALL cell lines (Fig. 3B). Based on these findings we conclude that DYRK1A regulates MYC levels independently of the *KMT2A* fusion oncogene. To determine if a regulatory feedback mechanism exists between DYRK1A and MYC, we analyzed DYRK1A expression levels at different stages during B cell development including transgenic mouse model systems with overexpression of *Bcl6* or *Myc/Bcl6* or deletion of *Lig4/tp53*, three distinct models of B cell lymphoma [40]. Notably, tumor cells isolated from the *Bcl6* transgenic and the *Myc/Bcl6* transgenic mice displayed reduced *Dyrk1a* mRNA expression levels (Fig. 3C and Supplementary Fig. 3A), indicating that Dyrk1a may be negatively regulated either by Bcl6 or Myc in these models.

### MYC, but not BCL6, negatively regulates DYRK1A

We next investigated whether BCL6 acts as a negative regulator of DYRK1A, given its role as a transcriptional repressor [41]. Using a meta-analysis of ChIP-Seq data from the ICN13 (KMT2A::AFF1) ALL PDX model, as well as the RS4;11 and SEM ALL cell lines (KMT2A::AFF1) [42], we found that BCL6 does not bind to the *DYRK1A* promoter (Supplementary Fig. 3B). Furthermore, genetic deletion of *Bcl6* in murine *Bcr-Abl1* ALL-like cells only resulted in moderate upregulation of *Dyrk1a* in one out of three probe sets (Supplementary Fig. 3C). Induced *Bcl6* deletion in an ALL-like mouse knockout model system with overexpression of *Tcf3-Pbx1* did not change RNA expression levels of DYRK1A, further validating that *BCL6* does not regulate *DYRK1A* transcript levels (Supplementary Fig. 3D). Overall, we conclude that Bcl6 does not directly regulate DYRK1A.

To then test if MYC regulates *DYRK1A* transcriptionally, we analyzed ChIP-Seq data of a human MYC-driven B cell malignancy model (P493-6) [43] in which *MYC* expression can be induced via removal of doxycycline from the cell culture medium [43]. Activation of MYC expression resulted in increased binding of MYC to the *DYRK1A* promoter in a time-dependent manner (Fig. 3D). To assess further if MYC negatively regulates DYRK1A, we performed loss-of-function analyses by examining gene expression data of P493-6 before and after 48 h of doxycycline-induced MYC deletion, which resulted in significant upregulation of DYRK1A (Fig. 3E). We validated MYC-mediated negative regulation of *Dyrk1a* using gene expression data of *Myc*-expressing and *Myc*-deleted *BCR-ABL1* transformed mouse ALL-like cells [44, 45] (Supplementary Fig. 4A). We also performed a gain-of-function meta-analysis of an Eμ-myc transgenic lymphoma/leukemia model [46, 47] (Fig. 3F) and observed that increased *Myc* levels correlate with decreased *Dyrk1a* levels during in vivo leukemia progression, further confirming a negative regulation of *Dyrk1a* via *Myc*.

Taken together, our analyses demonstrate that DYRK1A and MYC are closely connected and negatively regulate each other. *Dyrk1a* genetic deletion or DYRK1A pharmacologic inhibition resulted in increased Myc/MYC levels in our preclinical models, while genetic deletion or overexpression of *MYC* mediate increased or decreased *DYRK1A* expression, respectively.

### KMT2A-R ALL cells are sensitive to pharmacologic inhibition of DYRK1A

**Fig. 4:**
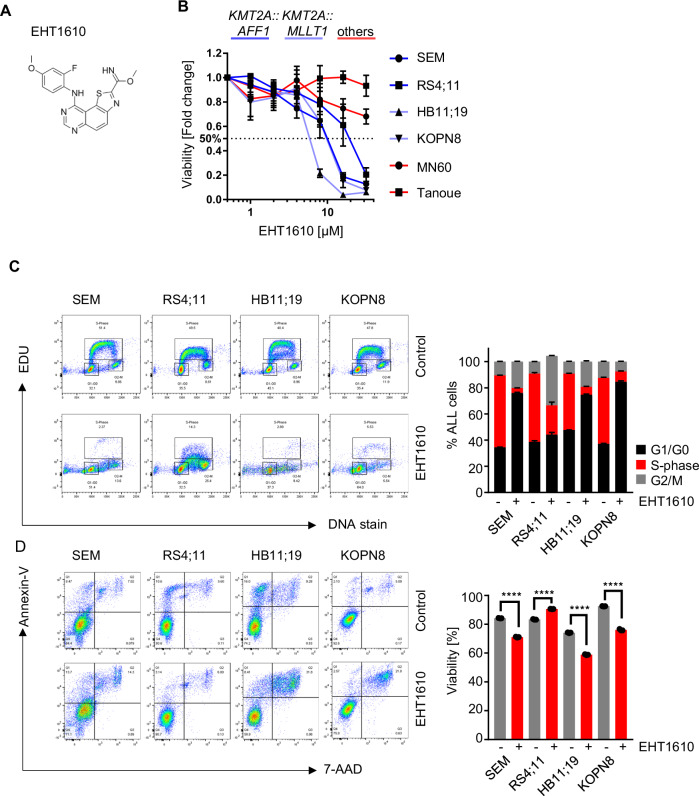
DYRK1A is required for *KMT2A*-R ALL cell proliferation. **A** Molecular structure of EHT161040. **B***KMT2A::AFF1* (dark blue; SEM and RS4;11), *KMT2A::MLLT1* (light blue; HB11;19 and KOPN8), and *non-KMT2A-R* ALL cell lines (red; MN60 and Tanoue) were treated with increasing concentrations of EHT161040. Viability/cell proliferation was determined via an XTT assay after 72 h. **C** Cell cycle analysis of the indicated *KMT2A*-R ALL cell lines after treatment with 5 µM EHT1610 for 72 h. On the left are representative examples of the flow cytometric analysis (*n* = 3), and on the right is the summary of all three experiments. **D** Cell viability and apoptosis were tested in control- and DYRK1A inhibitor-treated (EHT1610; 5 µM; 72 h) *KMT2A*-R ALL. On the left are representative examples of the flow cytometric analysis (*n* = 3) and on the right is the summary of all three experiments. Data are represented as individual values with mean ± SEM bars. *P < 0.05; **P < 0.01; ***P < 0.001 by *t*-test.

To evaluate DYRK1A as a potential target for the treatment of ALL, we first tested the extent to which in vitro pharmacologic inhibition of DYRK1A could induce apoptosis or cell cycle arrest in *KMT2A*-R ALL cells. Given that DYRK1A is important for Down Syndrome-associated ALL and the closely related *CRLF2*-R ALL [24, 25], we therefore excluded these ALL types from our analysis. We first used the DYRK1A inhibitor EHT1610 (Fig. 4A) for our translational studies. Interestingly, in vitro exposure to EHT1610 specifically reduced cell proliferation of *KMT2A*-R ALL compared to non-*KMT2A*-R ALL cell lines (Fig. 4B). It has been described in B- and T-cells that DYRK1A inhibition results in an accumulation of cells in the cell cycle S-phase [24]. To evaluate if DYRK1A inhibition affects cell proliferation or induces apoptosis specifically in *KMT2A*-R ALL, we performed a cell cycle analysis before and after EHT1610 treatment, which resulted in a significant reduction of cells in S-phase (Fig. 4C) and minimal effects upon cell viability (Fig. 4D). Taken together, DYRK1A inhibition in *KMT2A*-R ALL appears to affect cell proliferation specifically by increasing the number of cells in G1/G0 and limiting the number of cells in S-phase.

### Dual inhibition of DYRK1A and MYC increases apoptosis in certain KMT2A-R ALL cell lines but does not consistently enhance the therapeutic efficacy of DYRK1A inhibition

The role of MYC is highly complex, functioning as a well-known oncogene that drives cell proliferation and growth, while also paradoxically inducing apoptosis. In leukemia and certain solid tumors, MYC increases the expression of BIM, a proapoptotic protein, which can lead to cell death under specific conditions [48, 49]. Furthermore, elevated MYC levels can activate p53 [50], resulting in cell cycle arrest or apoptosis. Given the upregulation of MYC induced by DYRK1A inhibition, we investigated whether MYC upregulation would confer resistance to, or sensitize, *KMT2A*-R ALL cells to DYRK1A inhibition. Our flow cytometry (7-AAD/Annexin V) data indicate that dual inhibition of MYC (via the menin inhibitor VTP50469) and DYRK1A (EHT1610) significantly reduced viability in only one cell line (HB11;19) and had a moderate effect on KOPN8 cells, while other tested cell lines did not show increased cell death following dual DYRK1A and menin inhibition (Supplementary Fig. 2D). To confirm these findings, we conducted additional experiments using an MYC inhibitor (MYCi975) that prevents the dimerization of MYC and MAX, thereby blocking the expression of MYC target genes, including MYC itself [51, 52]. Western blot analysis confirmed the efficacy of MYCi975 in reducing MYC protein expression (Supplementary Fig. 2E). Additionally, we performed an XTT assay, which demonstrated that the response to dual treatment (MYC inhibitor + DYRK1A inhibitor) was consistent with the menin inhibitor data. Specifically, HB11;19 and KOPN8 were the only cell lines affected by the dual treatment (Supplementary Fig. 2F). These findings suggest that while MYC modulation can influence the response to DYRK1A inhibition in specific *KMT2A*-R ALL cell lines, its effects are not broadly consistent across all tested models, highlighting the complexity of MYC’s role in ALL.

### DYRK1A represses ERK signaling and protects KMT2A-R ALL cells from cell cycle arrest

**Fig. 5:**
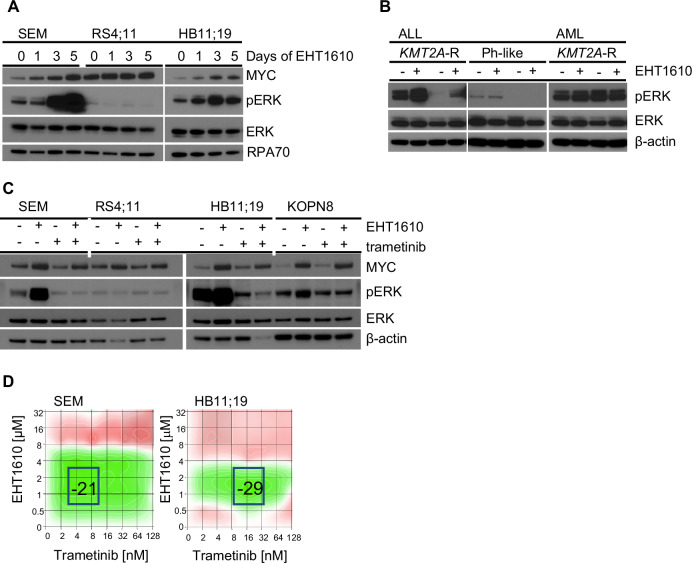
DYRK1A induces cell cycle arrest via ERK signaling pathway hyperactivation. **A** Protein expression levels of the indicated proteins were determined via Western blot using three *KMT2A*-R ALL cell lines treated with 5 µM EHT1610 for the indicated time points. **B** *KMT2A*-R (SEM and HB11;19) and Ph-like (MUTZ5 and MHH-CALL4) ALL cell lines as well as *KMT2A*-R (MOLM14, MV4;11) AML cell lines were treated either with control or 5 µM EHT1610. After 72 h pERK, ERK, and β-ACTIN protein levels were determined via Western blot. **C** The indicated *KMT2A*-R ALL cell lines were treated either with control, 5 µM EHT1610, 20 nM trametinib, or both drugs. After 72 h, the expression levels of the indicated proteins were determined via Western blotting. **D** The combinatorial effect of EHT1610 and trametinib was determined via synergy analyses using Synergy Finder software.

It has been demonstrated that DYRK1A overexpression results in increased ERK signaling output in brain cells and synovial tissues of rheumatoid arthritis patients [53–55]. Based on these findings, we hypothesized that the induction of cell cycle arrest in *KMT2A*-R ALL cells after pharmacologic inhibition may be via DYRK1A inhibitor-mediated downregulation of ERK signaling. To assess the effect of DYRK1A inhibition on ERK signaling, we treated different *KMT2A*-R ALL cell lines with EHT1610 and collected protein samples at four different time points. Surprisingly, we instead observed that DYRK1A inhibition resulted in hyperphosphorylation of ERK (Fig. 5A). To determine if this was a specific effect in *KMT2A*-R leukemias or if DYRK1A also mediates hyperphosphorylation in other ALL subtypes, we performed a Western blot to measure and compare ERK phosphorylation levels in vehicle- or EHT1610-treated *KMT2A*-R ALL and non-*KMT2A*-R ALL cell lines and in *KMT2A*-R AML cell lines. Interestingly, DYRK1A inhibitor-induced ERK hyperphosphorylation was restricted to *KMT2A*-R ALL samples (Fig. 5B). It has been demonstrated that hyperactivation of B cell receptor signaling molecules can result in negative selection of B cells [56] and that specifically the RAS/MEK/ERK signaling pathway is involved in this process [57]. Based on these findings, we hypothesized that DYRK1A inhibitor-mediated ERK hyperphosphorylation may result in negative selection and cell cycle arrest in *KMT2A*-R ALL cells. To test this hypothesis, we first treated 4 different *KMT2A*-R ALL cell lines either with vehicle control, EHT1610, the MEK1/2 inhibitor trametinib, or a combination of both drugs (Fig. 5C). As expected, DYRK1A inhibition resulted in potent ERK hyperphosphorylation in all tested *KMT2A*-R ALL cell lines with activated ERK signaling at a steady state, while trametinib inhibited ERK signaling. Most importantly, trametinib co-exposure effectively abrogated the EHT1610-mediated hyperphosphorylation of ERK. To test if trametinib would rescue *KMT2A*-R ALL cell proliferation, we performed a synergy study using increasing concentrations of EHT1610 and trametinib. Strikingly, our results demonstrate that trametinib rescued *KMT2A*-R ALL cells from DYRK1A inhibition-induced cell cycle arrest (Fig. 5D). Taken together, our results demonstrate an unexpected involvement of DYRK1A in RAS/MEK/ERK signaling regulation in *KMT2A*-R ALL cells and illustrate that DYRK1A inhibitor-mediated cell cycle arrest is induced via ERK hyperphosphorylation.

### DYRK1A inhibition induces the expression of BIM and reduces BCL-XL

**Fig. 6:**
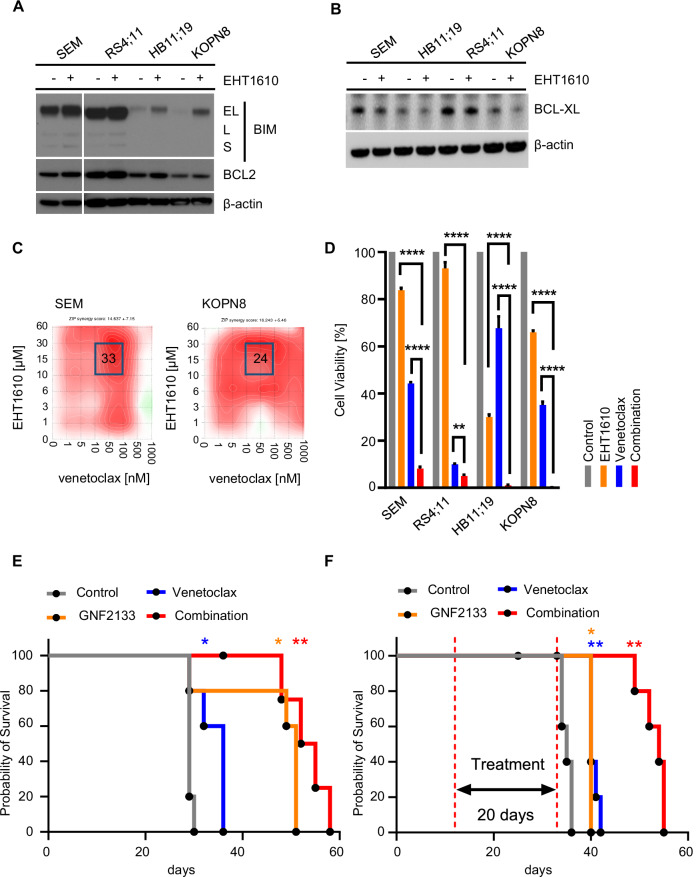
Dual DYRK1A and BCL2 inhibition synergistically kills *KMT2A-R* ALL. **A** Western blot analysis of the indicated *KMT2A*-R ALL cell lines treated either with vehicle control or 5 µM EHT1610. After 72 h, the expression levels of the indicated proteins were determined. **B** Western blot analysis of *KMT2A*-R ALL cell lines treated with control or 5 µM EHT1610. After 72 h, the expression levels of BCL-XL and β-actin were determined. **C** SEM and KOPN8 cells were treated with increasing concentrations of EHT1610 and venetoclax. The synergistic effect of both drugs was determined via Synergy Finder software as in Fig. 5. **D** Flow cytometric analysis of 4 *KMT2A*-R ALL cell lines treated with 5 µM EHT1610 and 20 nM venetoclax for 72 h. Shown is the statistical analysis (*n* = 3). **E** Kaplan–Meier analysis of the overall survival of mice injected with 1 million *KMT2A-AFF1* rearranged PDX cells (150MD) and treated either with control, GNF2133 (50 µM), venetoclax (50 µM), or a combination of both drugs. The treatment was started after ~5% CD19+/CD45+ALL cells were detected in the blood and continued until ~80% CD19+/CD45+ ALL replacements were detected in the blood of the mice. Significance was calculated via a log-rank test in Prism. **F** Kaplan–Meier survival analysis of *KMT2A::AFF1* ALL PDX model (ALL26MD) mice treated for 20 days with vehicle control, GNF2133 (50 µM), venetoclax (50 µM), or a combination of both drugs (*n* = 5 mice/cohort). Significance was calculated via a log-rank test in Prism. Data are represented as individual values with mean ± SEM bars. *P < 0.05; **P < 0.01; ***P < 0.001 by *t*-test or Log-rank (Mantel–Cox) test.

The proapoptotic factor BIM is regulated via multiple regulatory mechanisms. One of these regulators is MYC, which has been demonstrated to directly bind to the BIM promoter region to activate BIM-mediated apoptosis [49]. ERK has also been studied as a molecule that can either increase the proapoptotic activity via phosphorylation of BIM at S44, T56, and S58 residues (exon 3) [58] or decrease its protein stability and induce proteasome-dependent degradation via phosphorylation of S69 [59, 60]. To ascertain if DYRK1A inhibition and consequent activation of MYC and ERK signaling results in increased BIM expression and activity in *KMT2A*-R ALL cells, we treated four cell lines with EHT1610 and performed a Western blot analysis of BIM and BCL2. The proapoptotic activity of BIM is neutralized when BCL2 binds to BIM [61]. Strikingly, DYRK1A inhibition increased BIM expression in three of the four *KMT2A*-R ALL cell lines, while the BCL2 levels remained mostly unchanged (Fig. 6A). We validated that BIM activation starts as early as 24 h (Supplementary Fig. 4C). Furthermore, DYRK1A inhibition resulted in the reduction of the antiapoptotic factor BCL-XL, further indicating that DYRK1A inhibition renders cells sensitive to apoptosis (Fig. 6B). Taken together, these data indicate that DYRK1A inhibition may induce apoptosis via upregulation of BIM and downregulation of BCL-XL.

### *DYRK1A inhibition renders cells sensitive to venetoclax* and increases overall survival of mice in preclinical in vivo studies

To determine if DYRK1A inhibition sensitizes *KMT2A*-R ALL cells to BCL2 inhibition, we treated *KMT2A*-R ALL cells in vitro with increasing concentrations of EHT1610 and the BCL2 inhibitor venetoclax and observed synergistic killing (Fig. 6C) and decreased cell viability, as assessed by flow cytometry (Fig. 6D and Supplementary Fig. 4B).

Given our observations of significant EHT1610-associated toxicity in mice, we synthesized the recently described DYRK1A inhibitor GNF2133 (Supplementary Fig. 5A), which has been shown to be well tolerated in vivo in mice and rats in preclinical studies [62]. First, we compared GNF2133 to EHT1610 and their ability in vitro to induce ERK hyperphosphorylation in *KMT2A-*R ALL cells (Supplementary Fig. 5B) and confirmed that GNF2133 exposure decreased the percentage of cells in S-phase compared to control treatment (Supplementary Fig. 5C). We further observed synergistic killing of *KMT2A*-R ALL cell lines when co-treated with GNF2133 and venetoclax (Supplementary Fig. 5D, E) and GNF2133-mediated ERK hyperphosphorylation in *KMT2A*-R PDX cases (Supplementary Fig. 5F).

We then confirmed in a pilot feasibility study that optimized GNF2133 doses of up to 50 mg/kg daily ×14 days in vivo did not induce untoward toxicity in NSG mice (data not shown). *KMT2A*-R ALL PDX model (ALL150MD) mice were then treated with vehicle control, GNF2133 (50 mg/kg), venetoclax (50 mg/kg), or both inhibitors. Single-agent GNF2133 or venetoclax each reduced leukemia burden, but when GNF2133 and venetoclax were given in combination, the leukemic burden was further reduced (Supplementary Fig. 6A) and prolonged animal survival (Fig. 6E). Dual inhibitor therapy again appeared tolerable in vivo given weight stability of treated mice over time (Supplementary Fig. 6B). The criteria for mice to reach the study endpoint included either >80% CD19+/CD45+ cells in the blood, 20% weight loss, and/or reduced physical mobility due to leukemia progression or potential drug toxicity as described [28, 29, 63]. Once any of these criteria were met, the mice were sacrificed. Supplementary Fig. 6C shows that, at the time of sacrifice, the spleens were equally enlarged across all treatment groups. Results were validated in a second *KMT2A*-R ALL PDX model (ALL26MD) with the greatest inhibition of leukemia proliferation (Supplementary Fig. 6D), improved survival (Fig. 6F), and tolerability (Supplementary Fig. 6E) observed with dual GNF2133 and venetoclax treatment. Furthermore, spleen enlargement was equal among the different treatment groups (Supplementary Fig. 6F).

To test whether DYRK1A inhibition affects healthy hematopoietic cells, we treated C57BL/6 mice for two weeks with either vehicle control or GNF2133 (50 mg/kg) and subsequently performed a complete blood count (CBC). The results show no significant changes in hematopoietic cell populations between the control and GNF2133-treated mice, except for a reduction in eosinophils and basophils following GNF2133 treatment (Supplementary Fig. 7A, B). During the two-week treatment period, none of the mice experienced significant weight loss (Supplementary Fig. 7C).

Taken together, these data credential the combined inhibition of DYRK1A and BCL2 as an additional effective and potentially clinically translatable therapeutic strategy for *KMT2A*-R ALL.

## Discussion

To identify new targets in *KMT2A*-R ALL for precision medicine approaches, we performed a kinome-wide CRISPR screen and identified multiple kinases, including DYRK1A, as required for leukemia cell survival. We selected to further study the importance of DYRK1A for *KMT2A*-R ALL as it met the following three criteria: (1) Growth inhibition upon DYRK1A targeting was stronger in *KMT2A*-R leukemic cells as in non-*KMT2A*-R ALL cells, (2) DYRK1A is not a common essential gene assessed via the Cancer Dependency MAP, and (3) DYRK1A has not been studied specifically in *KMT2A*-R ALL. Previous studies have suggested the involvement of DYRK1A in Down syndrome-associated megakaryoblastic leukemia [23] and normal hematopoiesis [24, 64]. More recently, additional studies support a critical role of DYRK1A in DS-ALL [25] and the closely related *CRLF2-*R Ph-like ALL subtype [25]. While higher DYRK1A expression levels are expected in ALL cells from patients with Down syndrome, given that DYRK1A is located on chromosome 21, it is not known how DYRK1A is transcriptionally regulated. Here we demonstrate a direct transcriptional regulation of DYRK1A via *KMT2A* fusions in ALL. In addition, we report that pharmacologic inhibition of menin, an essential transcription factor involved in the regulation of *KMT2A*-R target genes [65–67], and direct DYRK1A inhibition results in cell cycle arrest of *KMT2A*-R ALL. Given that *DYRK1A* is not the only target gene regulated by *KMT2A*, we specifically focused on studying the importance of DYRK1A for *KMT2A*-R ALL survival using DYRK1A inhibitors. We specifically tested two DYRK1A inhibitors (EHT1610 and GNF2133) for our studies, which are both specifically inhibiting DYRK1A with very little off-target effects. However, in our in vivo studies EHT1610 treatments were toxic to the recipient mice resulting in a premature ending of the study. We attribute the toxicity observed with EHT1610 in part to incomplete solubilization as evidenced by peritoneal crystallization of the compound resulting in toxicity and likely decreased bioavailability. Batch variability and long-term storage of lyophilized EHT1610 may contribute to inconsistent solubilization; a recurrent obstacle in developing DYRK1A-selective ATP-competitive inhibitors has been poor aqueous solubility [68]. Using the DYRK1A inhibitor GNF2133 was significantly better tolerated by the mice based on our data as well as published data [62]. In previous experiments, we also tested harmine, a widely used DYRK1A inhibitor, to examine the combinatorial effect of dual DYRK1A and BCL2 inhibition. Similar to GNF2133, harmine treatment in combination with venetoclax led to a significant reduction in *KMT2A*-R ALL in mice, thereby validating our findings and underscoring DYRK1A’s critical role in *KMT2A*-R ALL (data not shown). However, harmine’s application in humans is limited due to associated toxicities, particularly neurotoxicity. Thus, the in vivo efficacy of GNF2133 becomes particularly valuable and highlights the need for further development of pharmacologic agents targeting DYRK1A.

Our results demonstrate that *KMT2A*-R ALL requires DYRK1A for normal cell proliferation and dual inhibition of DYRK1A and BCL2 decreases leukemia progression in *KMT2A*-R ALL PDX models.

ALL cells are bone marrow-derived B cell precursors that have lost the ability to differentiate but retain some properties of B cells. In B cells, it has been shown that the PI3K and ERK signaling pathways are regulated via the B cell receptor, and depending on the signaling strength, may mediate negative selection to prevent autoimmunity. Previous results in normal B cells have suggested that DYRK1A regulates B cell differentiation [24]. Additionally, it has been demonstrated that DYRK1A regulates B cell survival and B cell autoimmunity via activation of NF-κB [64]. Our results demonstrate that DYRK1A inhibition in *KMT2A*-R ALL prevented cell proliferation while interestingly resulting in hyperphosphorylation of ERK, in contrast to prior reports in other cell types [53, 55]. Dual inhibition of DYRK1A and the ERK regulating kinases MEK1/2 prevented ERK hyperphosphorylation and consequently rescued cell proliferation. demonstrating that DYRK1A-mediated hyperphosphorylation of ERK is specifically negatively affecting cell proliferation and survival. Given that *KMT2A*-R ALL cells do not express a (pre) B cell receptor on the surface, we propose that the mechanism of negative selection is induced independently of the B cell receptor in these cells and that DYRK1A downregulates the negative selection of *KMT2A*-R B-ALL, thereby facilitating rapid leukemia cell proliferation. Upon DYRK1A inhibition, ERK hyperphosphorylation may stimulate a type of negative selection that makes cells vulnerable to inhibition of BH3-based cell survival mechanisms.

Another important observation is that DYRK1A and MYC negatively regulate each other in a feedback loop. First, we identified that pharmacologic DYRK1A inhibition resulted in increased MYC levels via decreased phosphorylation of Thr58 and Ser62 on MYC, which is concordant with a recent publication demonstrating that viral overexpression of DYRK1A in non-*KMT2A*-R AML results in phosphorylation of Thr58 and Ser62 on MYC and consequently in MYC degradation [38]. A critical regulator of apoptosis is BIM, which is directly regulated via MYC. Per our results, DYRK1A inhibition results in upregulation of MYC and consequently in higher BIM levels, while the BIM negative regulator BCL2 is not affected. Furthermore, we demonstrate that upon DYRK1A inhibition, the expression levels of the antiapoptotic factor BCL-XL are reduced, which consequently renders *KMT2A*-R ALL cells sensitive to dual DYRK1A and venetoclax inhibition and significantly extends the survival of mice transplanted with *KMT2A*-R ALL, indicating that a DYRK1A inhibitor-based therapy is a novel approach for the treatment of *KMT2A-*R ALL.

Despite significant improvements in the treatment of patients with acute leukemias, including improved access to relevant targeted inhibitors and antibody-based or cellular immunotherapies, children and adults with *KMT2A*-R ALL continue to experience inferior clinical outcomes. Our preclinical data suggest that co-targeting of DYRK1A and menin or BCL2 has particular activity against this high-risk leukemia subtype that may warrant clinical investigation in patients in the future to address issues of chemoresistance and relapse risk.

## Supplementary information

Supplementary Information

## Data Availability

All data used in this study are publicly available and can be accessed from the Gene Expression Omnibus: GSE83671, GSE38403, GSE84116, GSE127508, GSE26408, GSE36354, and GSE120246.
