# Supplementary material for: DYRK1A inhibition results in MYC and ERK activation rendering KMT2A-R acute lymphoblastic leukemia cells sensitive to BCL2 inhibition
Source: Leukemia. 2025 Mar 27;39(5):1078–89. doi: 10.1038/s41375-025-02575-w (PMC12055583; doi:10.1038/s41375-025-02575-w)
Supplement: Supplementary file 1 — Supplementary Information [file 41375_2025_2575_MOESM1_ESM.docx]

**DYRK1A Inhibition Results in MYC and ERK Activation Rendering *KMT2A*-R Acute Lymphoblastic Leukemia Cells Sensitive to BCL2 Inhibition**

**Extended Method Section:**

***Pooled CRISPR screening***

The kinase domain-focused sgRNA library screening has been performed as previously described^1^. To perform the kinome-wide kinase sgRNA screen, we first virally transformed SEM, HAL-01, and TVA-1 cells with a LentiV_Cas9_puro vector^1^. After successful puromycin selection, we then further transduced the ALL cells with lentivirus of pooled sgRNA library as described. Virus titer was measured by infection of cells with serially diluted virus. For the transduction of single sgRNA per cell, the multiplicity of infection (MOI) was set to 0.3-0.4. To maintain the representation of sgRNAs during the screen, the number of cells was kept 1000 times more than the sgRNA number in the library. Cells were harvested at the initial (day 3 post-infection) and final (21 days after initial) timepoints. Genomic DNA was extracted using QIAamp DNA mini kit (QIAGEN). Analysis was performed as previously described^2^.

The secondary reduced library screen was performed on SEM cells as described above. The reduced library included 14 kinases that we have identified in our primary screen to be required for *KMT2A*-R ALL survival and 21 negative controls (**Table S4)**.

***Patient-derived xenograft (PDX) modeling and in vivo preclinical drug trials***

ALL PDX model establishment and experimental animal studies were conducted on Institutional Animal Care and Use Committee (IACUC)-approved research protocols at CHOP or Temple University in accordance with NIH and American Veterinary Association Guidelines for the Euthanasia of Animals. Briefly, *KMT2A*-R and non-*KMT2A*-R ALL PDX were created via intravenous injection of primary human ALL cells into 6–8-week-old male and female NOD/SCID/IL-2rγ^null^ (NSG), mice as previously described^3-6^. After flow cytometric confirmation of $\geq$1% CD45+ CD19+ human ALL in murine peripheral blood, animals were randomized to treatment with indicated combinations of (1) vehicle via oral gavage (PO) or intraperitoneal (IP) injection, (2) GNF2133 at 50 mg/kg IP, (3) venetoclax 50 mg/kg PO, (4) a combination of both drugs for up to 20 days depending on rate of leukemia progression in control animals requiring study termination at a pre-determined endpoint per our established methods (5 mice/group)^3, 4, 7^. Animals were excluded from the study if the cause of death was unrelated to leukemia. The studies were performed in a non-blinded manner.

***Flow cytometry analysis***

Human and mouse leukemia samples were stained with cell surface antibodies (**Table S5**) according to the manufacturer's instructions using Fc block and respective isotype controls. Annexin V, propidium iodide, 7-AAD, or DAPI (BD Biosciences) were used for apoptosis analyses (**Table S5**). Flow cytometry was performed using FACSVerse or LSRII flow cytometers (BD Biosciences) with data analysis in Cytobank (Beckman-Coulter; Palo Alto, California) or FlowJo (TreeStar; Ashland, Oregon) and graphical display and statistical analysis in Prism (GraphPad; La Jolla, California).

***Western Blotting***

All cells were lysed in CelLytic buffer (Sigma; Burlington, MA) supplemented with 1% HALT protease inhibitor and phosphatase inhibitor cocktail (Thermo Scientific; Waltham, MA). Protein samples were loaded on NuPAGE 4-12% Bis-Tris gradient gels (Invitrogen) and transferred on to PVDF membranes (Millipore; Burlington, MA). For the detection of mouse and human proteins, primary antibodies were used in combination with anti-rabbit or anti-mouse HRP-linked secondary antibodies (Cell Signaling; Danvers, MA) and Amersham ECL Western blotting detection reagent (GE Life Sciences; Marlborough, MA; **Table S5**).

***Cell proliferation and viability assays***

One hundred thousand human ALL cells were seeded in a volume of 100 μL B cell medium/well (as described above) on Optilux 96-well plate (BD Biosciences, San Jose, CA). EHT1610, and venetoclax were diluted in medium and added at the indicated concentration in a total culture volume of 150 μL. MI-503 was synthesized by Wuxi Pharma as previously described^8-10^. After culturing for 72 hours, cell proliferation and viability were measured by XTT (Cell Signaling) according to the manufacturer’s instructions with fluorescence read at 450 nm. Fold changes were calculated using baseline values of untreated cells as a reference (set to 100%) and displayed graphically in Prism.

***Quantitative real-time polymerase chain reaction (qRT-PCR)***

Total RNA from leukemia cells was extracted using RNeasy isolation kit (Qiagen). cDNA was generated using the qScript cDNA SuperMix (Quanta). Quantitative real-time PCR was performed with Power SYBR® Green Master Mix (Applied Biosystems) and 7500 Real Time PCR System (Applied Biosystems) according to standard PCR conditions. Primers for quantitative RT-PCR are listed in **Table S6.**

***In vitro drug synergy analysis***

To perform the synergy experiments, cells were treated with increasing concentrations of the tested drugs for 72h. After 72h, cell viability was analyzed via XTT assay. The expected drug combination responses between EHT1610 and trametinib or EHT1610 and venetoclax were calculated based on the ZIP reference model using SynergyFinder^11^. Deviations between observed and expected responses with positive and negative values denote synergy and antagonism, respectively. For the estimation of outlier measurements, cNMF algorithm^12^ implemented in SynergyFinder was utilized.

***Statistical analyses***

Data display and statistical analyses were performed using Prism 7 (GraphPad). Significance values for *in vitro* and *in vivo* studies were calculated using unpaired two-tailed student t-test (2 groups) or ANOVA with Dunnett’s or Tukey’s post-test for multiple comparisons (3 or more groups) to determine statistical significance. Survival analysis distribution between 2 groups for clinical patient samples was determined by the log-rank test. A *P* value of less than 0.05 was considered significant. All values are expressed as mean ± SEM.

***Synthesis of GNF2133***

GNF2133 was synthesized in nine steps using the method previously described by Liu et al.,.^13^

**
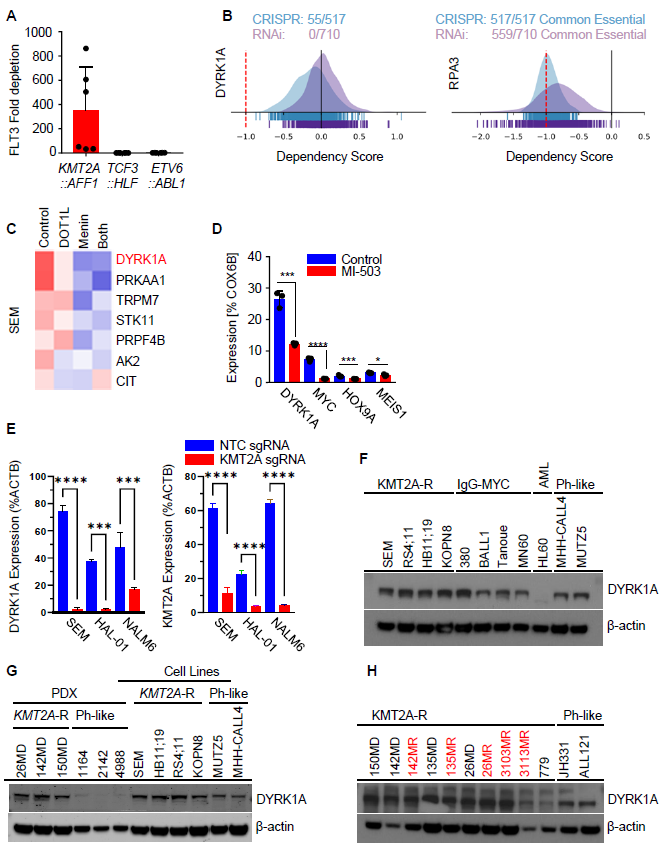
**

**Supplementary Figure 1**

**A)** Shown is the fold depletion of FLT3 among the different ALL cell lines used for the kinome-wide CRISPR screen (shRNAs, n=6). **B)** Dependency analysis of DYRK1A and RPA3 via the Cancer Dependency MAP website. **C)** Analysis of gene expression data from SEM cells that were treated with DMSO, 3 μM EPZ004777 (DOT1L inhibitor), 3 µM MI-2-2 (menin inhibitor), or a combination of both for 4 days as previously published (GSE63664)^14^. Shown is a list of target genes that were identified in our kinome-wide CRISPR screen. **D)** RT-PCR was performed to determine the gene expression levels of the indicated genes in SEM cells after 5 days of treatment either with control or 3 µM MI-503 (menin inhibitor **E)** Electroporation of nontargeting (NT) crRNAs or crRNAs targeting KMT2A, was performed to transfect Cas9-transduced *KMT2A*-R (SEM) and non-*KMT2A*-R (HAL-01, NALM6) ALL cells. The efficiency of CRISPR/Cas9-mediated deletion of KMT2A was evaluated through RT-PCR analysis, alongside assessments of DYRK1A expression levels. **F)** Western blot analysis of the indicated cell lines for the protein expression levels of DYRK1A and β-actin. **G)** Western blot analysis of DYRK1A and β-actin in *KMT2A*-R and Ph-like PDX cases as well as *KMT2A*-R and Ph-like ALL cell lines. **H)** The protein levels of DYRK1A and β-actin were asessed via Western blotting of cells harvested from paired diagnosis (black) and relapse (red) *KMT2A*-R ALL PDX models; two Ph-like ALL PDX cases were used as controls. Data are represented as individual values with mean ± SEM bars by test.

**
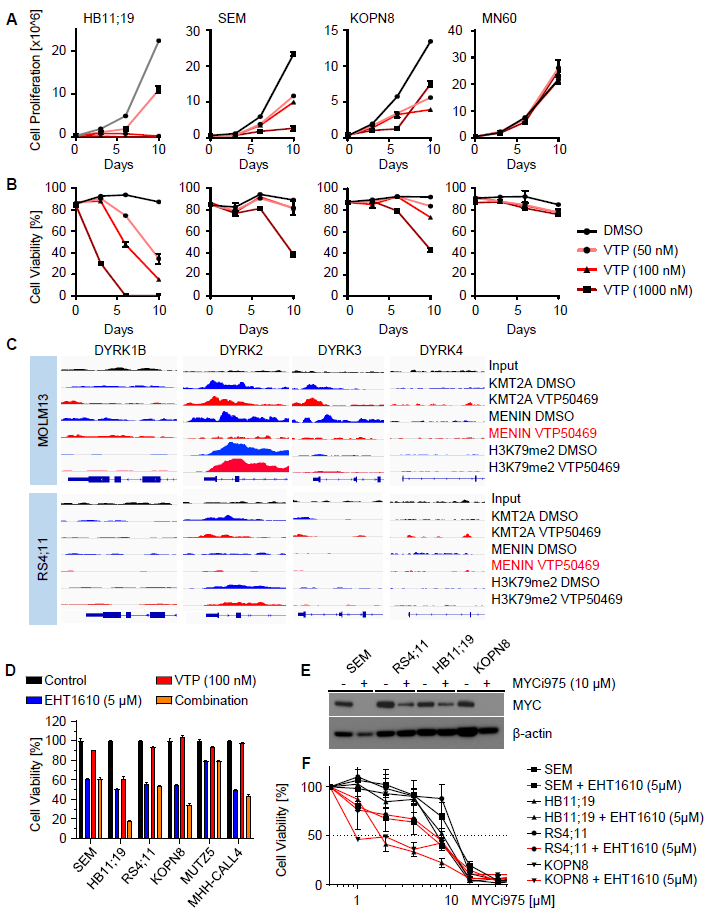
**

**Supplementary Figure 2**

The *KMT2A*-R ALL cell lines HB11;19, SEM, KOPN8, and the non-*KMT2A*-R ALL cell line MN60 were treated with three different concentrations of the menin inhibitor VTP50469 (50 nM, 100 nM, 1000 nM) for the indicated time points. Cell number **(A)** and cell viability **(B)** were determined via the autonomous cell counter system (n=3). **C)** Analysis of ChIP-Seq tracks on DYRK family member promoter regions using *KMT2A* N-terminal-, menin-, and H3K79me2-specific antibodies. Two *KMT2A*-R cell lines (MOLM13 [AML] and RS4;11 [ALL]) were either treated with veichle control or the menin inhibitor VTP50469 as previously described (GSE127508)^15^. **D)** 7-AAD flow cytometry-based viability analysis of *KMT2A*-R ALL cell lines (SEM, HB11;19, RS4;11, KOPN8) and Ph-like ALL cell lines (MUTZ5, MHH-CALL4) treated for 72 hours with either control, EHT1610 (5 µM), VTP50469 (100 nM), or a combination of both drugs. **E)** Western blot analysis of the indicated cell lines after treatment with MYCi975 (10 µM) for 48 hours. **F)** XTT analysis of the indicated cell lines treated with either a control or 5 µM EHT1610, along with increasing concentrations of MYCi975, for 72 hours.


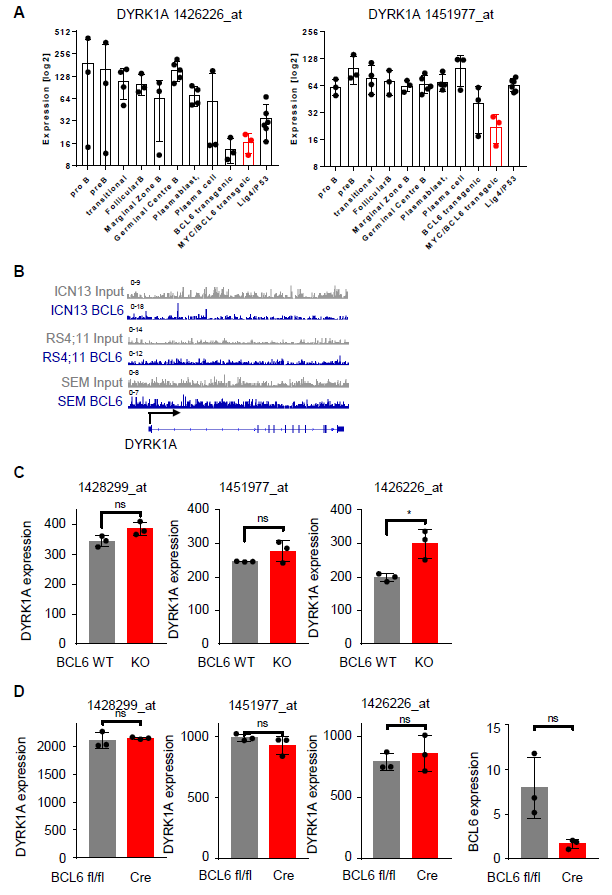


**Supplementary Figure 3**

**A)** DYRK1A gene expression levels in a comprehensive panel of purified developmentally defined normal murine B cells and genetically distinct murine lymphoma models (GSE26408)^16^. Shown are the probe sets 1426226_at and 1451977_at. **B)** BCL6 ChIP-Seq analysis of harvested ALL cells from PDX model ICN13 (*KMT2A::AFF1*) and ALL cell lines (RS4;11, *KMT2A::AFF1*; SEM, *KMT2A*::*AFF1*; GSE59541)^17^. **C)** Gene expression analyses of BCL6 WT and KO mouse ALL cells. Shown are three different probe sets for DYRK1A (GSE20987)^18^. **D)** Gene expression analyses of conditional BCL6^fl/fl^ mouse ALL cells. Shown are three different probe sets for DYRK1A and one for BCL6. Deletion of BCL6 was induced via CRE as previously published (GSE59332)^19^. Data are represented as individual values with mean ± SEM bars by test.


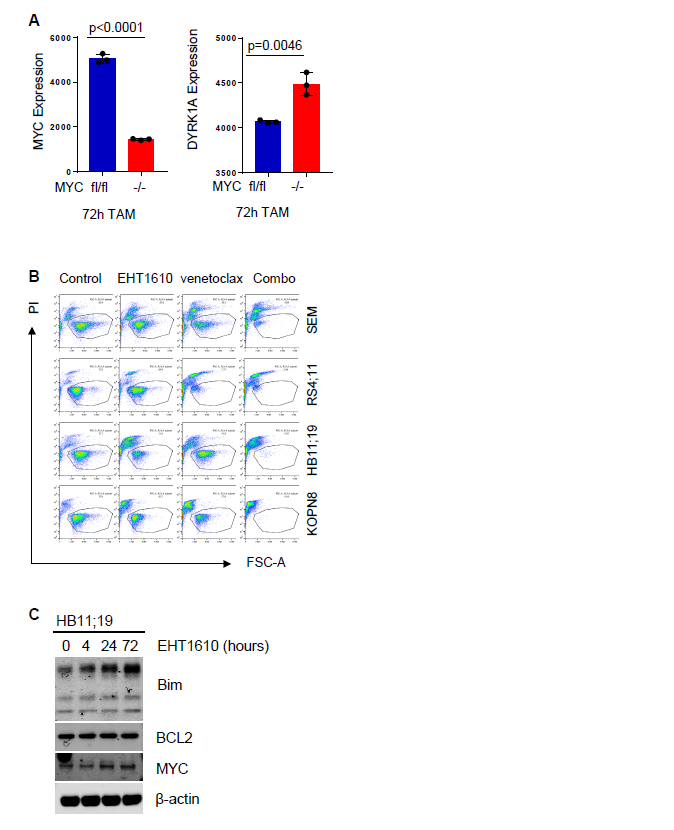


**Supplementary Figure 4**

**A)** Gene expression analyses of conditional MYC^fl/fl^ mouse BCR-ABL1 transduced ALL cells. Shown are the gene expression levels of MYC and DYRK1A. Deletion of MYC was induced via CRE as previously published (GSE30928)^20^. **B)** 4 *KMT2A*-R ALL cells were treated with 5 µM EHT1610 and 20 nM venetoclax for 72h. Shown are representable examples of the flow cytometric viability analysis (n=3). Data are represented as individual values with mean ± SEM bars by test. **C)** SEM cells were treated with 5 µM EHT1610 at 0, 4, 24, and 72 hours before protein isolation. Western blot analysis was performed for the indicated proteins.

**
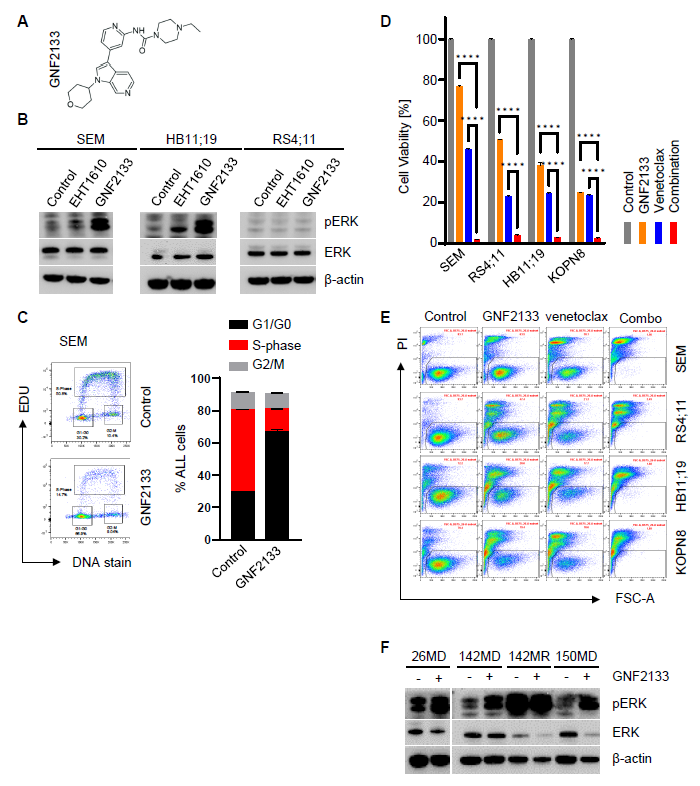
**

**Supplementary Figure 5**

**A)** Molecular structure of GNF2133^21^. **B)** Western blot analysis of *KMT2A*-R ALL cell lines treated with EHT1610, GNF2133 (5 µM) for 72h for the indicated proteins. **C)** Cell cycle analysis (Edu) of SEM cells treated with 5 µM GNF2133 for 72h. Shown are examples of FACS plots (left) and statistical analysis (right; n=3). **D/E)** 4 *KMT2A*-R ALL cell lines were treated with 5 µM GNF2133 and 20 nM venetoclax for 72h. Shown are the statistical analysis (D) and representable examples of the flow cytometric viability analysis (E; n=3). **F)** Western blot analysis of 4 *KMT2A*-R ALL PDX model cells treated *in vitro* with GNF2133 (5 µM) for 72h for the indicated proteins. Data are represented as individual values with mean ± SEM bars. *P < 0.05; **P < 0.01; ***P < 0.001 by t-test.


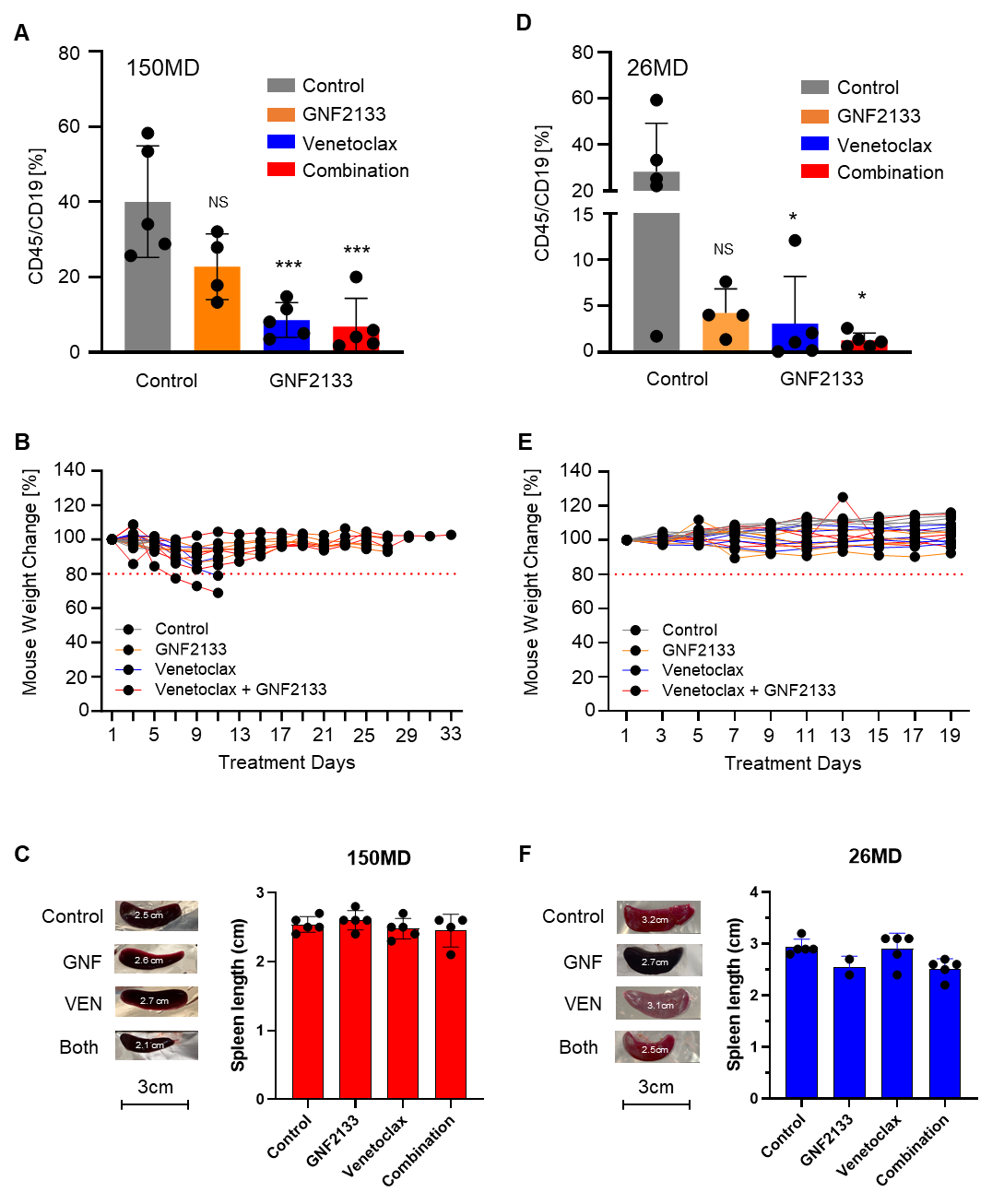


**Supplementary Figure 6**

**A)** Flow cytometric analysis of CD19+/CD45+ ALL progression in the blood of mice injected with *KMT2A*-R ALL PDX cells (model ALL150MD^22, 23^) after 1 week of treatments (n=5). **B)** The weights of mice in different treatment groups were monitored throughout the treatment to test for drug-associated toxicities. **C)** The spleens of the mice were isolated after sacrifice, and spleen sizes were plotted for each treatment group. A representative image of a spleen from each treatment group is shown. **D)** Flow cytometric analysis of CD19+/CD45+ ALL progression in the blood of mice injected with *KMT2A*-R ALL PDX cells (model ALL26MD) after 1 week of treatments (n=5). **E)** The weights of mice in different treatment groups were monitored throughout the treatment to test for drug-associated toxicities. **F)** The spleens of the mice were isolated after sacrifice, and spleen sizes were plotted for each treatment group. A representative image of a spleen from each treatment group is shown. Data are represented as individual values with mean ± SEM bars. *P < 0.05; **P < 0.01; ***P < 0.001 by 2-way ANOVA with Tukey’s multiple comparisons test.

**
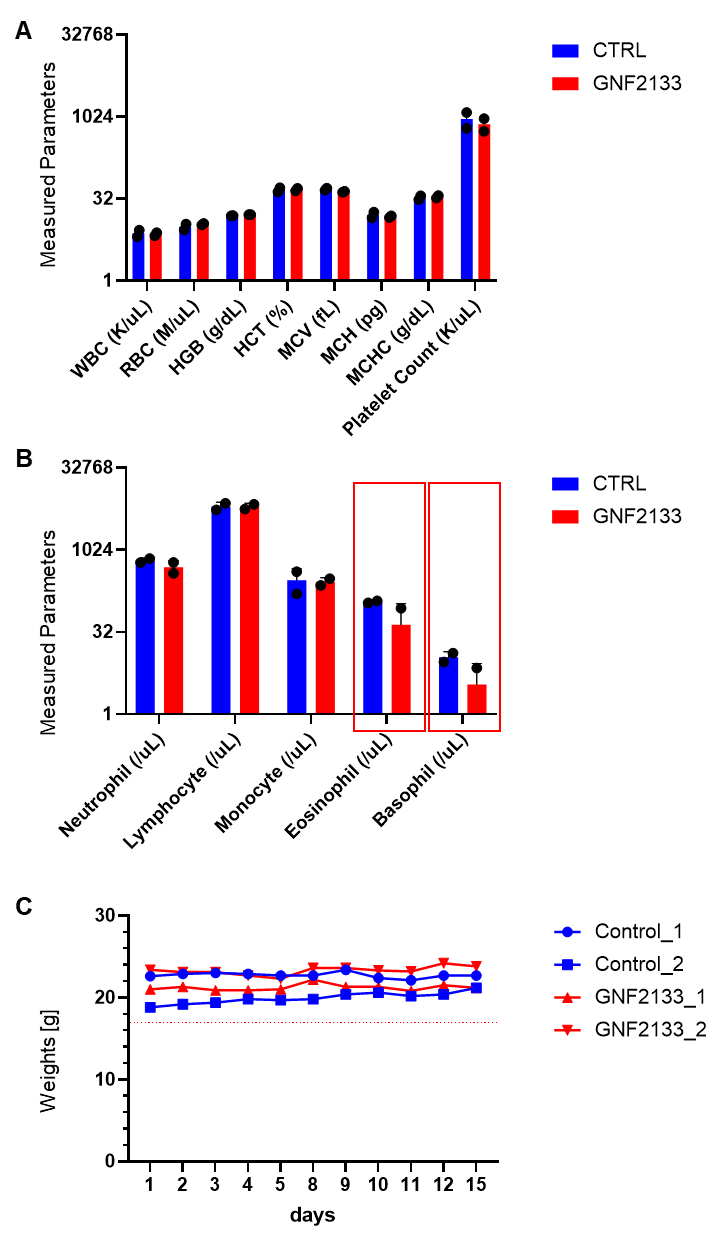
**

**Supplementary Figure 7**

Two C57BL/6J mice were treated for 2 weeks with GNF2133 (50mg/kg). After the last treatment, blood was isolated from the mice and a complete blood count (CBC) testing was performed. **A/B**) shows the result of the CBC testing. **C)** during the two week treatment the weight of the mice was monitored.

**Supplementary Table 1. ALL PDX models used in these studies.** Models were previously described in publications^23, 24^.

| **PDX model** | **Genetic alteration** | **FLT3/RAS status** | **Other Know Mutations** | **Diagnosis/Relapse** |
| --- | --- | --- | --- | --- |
| ALL150MD | *KMT2A::AFF1* | *KRAS*-mutant |  | diagnosis |
| ALL142MD | *KMT2A::AFF1* | *NRAS*-mutant |  | diagnosis |
| ALL142MR | *KMT2A::AFF1* | *NRAS*-mutant | *IKZF1* del, cnLOH of chr22 | relapse |
| ALL135MD | *KMT2A::MLLT1* |  |  | diagnosis |
| ALL135MR | *KMT2A::MLLT1* |  |  | relapse |
| 2ALL6MD | *KMT2A::MLLT1* |  |  | diagnosis |
| ALL26MR | *KMT2A::MLLT1* |  | Partial 10q del, including *PTEN* | relapse |
| ALL3103 | *KMT2A::MLLT1*3 |  |  | relapse |
| UP_ALL3113 | *KMT2A::AFF1* |  | *JAK2*-mutant, *TP53*-deleted, *IKZF1*-deleted | diagnosis |
| UP_ALL779 | *KMT2A::AFF1* |  |  | diagnosis |
| TVA1 | *ETV6::ABL1* |  |  | diagnosis |
| JH331 | *IGH::CRLF2* |  |  | diagnosis |
| ALL121 | *IGH::CRLF2* |  |  | relapse |
| 1164 | *P2RY8::CRLF2* |  | *JAK2*-mutant |  |
| 2142 | *IGH::CRLF2* |  |  |  |
| 4988 | *IGH::CRLF2* |  | *JAK2*-mutant |  |

**Supplementary Table 2. Leukemia cell lines used in these studies.**

| **Cell Line** | **Lesion** | **RAS status** | **Leukemia type** | **Source** |
| --- | --- | --- | --- | --- |
| SEM | *KMT2A::AFF1* | *FLT3* intrachromosomal gene amplification | B-ALL | DSMZ |
| RS4;11 | *KMT2A::AFF1* |  | B-ALL | DSMZ |
| HB11;19 | *KMT2A::MLLT1* | *FLT3*-mutant | B-ALL | Dr Patrick Brown, Johns Hopkins University |
| KOPN8 | *KMT2A::MLLT1* | *KRAS*-mutant | B-ALL | DSMZ |
| HAL-01 | *TCF3::HLF* |  | B-ALL | DSMZ |
| MN60 | *MYC::IGH* |  | B-ALL | DSMZ |
| Tanuoe | *MYC::IGH* |  | B-ALL | DSMZ |
| 308 | *MYC::IGH* |  | B-ALL | DSMZ |
| BALL1 | *MYC::IGH* |  | B-ALL | DSMZ |
| MUTZ5 | *IGH::CRLF2* |  | B-ALL | DSMZ |
| MHH-CALL4 | *IGH::CRLF2* | *KRAS*-mutant | B-ALL | DSMZ |
| HL60 | *PML::RARA* |  | AML | DSMZ |
| MOLM14 | *KMT2A::MLLT1* | *FLT3*-ITD | AML | DSMZ |
| MV4;11 | *KMT2A::MLLT1* | *FLT3*-ITD | AML | DSMZ |

**Supplementary Table 3. Viral Constructs**

| **Viral Construct** | **Insert** | **Purpose** |
| --- | --- | --- |
| pCL6-LUC-Blast | luciferase | Detection of human leukemic cells in vivo |
| LentiV-CAS9-Puro | CAS9 | CRISPR-CAS9 mediated deletion of target genes |
| LRG2.1-GFP | CRISPR-kinase library | Kinome wide CRISPR Screen |
| LRG2.1-GFP | reduced CRISPR-kinase library | Deletion of specific kinases |

**Supplementary Table 4. Reduced CRISPR Library**

| **Sequence Name** | **Sequence** |
| --- | --- |
| DYRK1A_PKc_E5_148 | CAC CGT GTA AAG GCA TAT GAT CGT G |
| DYRK1A_PKc_E7_147.1 | CAC CGT CGC TTT TAT CGG TCT CCA G |
| neg01 | CAC CGT AGC GAA CGT GTC CGG CGT |
| neg02 | CAC CGA CCG GAA CGA TCT CGC GTA |
| neg03 | CAC CGG CAG TCG TTC GGT TGA TAT |
| neg04 | CAC CGC TTG AGC ACA TAC GCG AAT |
| neg05 | CAC CGT GGT AGA ATA ACG TAT TAC |
| neg06 | CAC CGT CAT ACA TGG ATA AGG CTA |
| neg07 | CAC CGA TAC ACG AAG CAT CAC TAG |
| neg08 | CAC CGA ACG TTG GCA CTA CTT CAC |
| neg09 | CAC CGA TCC ATG TAA TGC GTT CGA |
| neg10 | CAC CGT CGT GAA GTG CAT TCG ATC |
| neg11 | CAC CGT TCG ACT CGC GTG ACC GTA |
| neg12 | CAC CGA ATC TAC CGC AGC GGT TCG |
| neg13 | CAC CGA AGT GAC GTC GAT TCG ATA |
| neg14 | CAC CGC GGT GTA TGA CAA CCG CCG |
| neg15 | CAC CGT ACC GCG CCT GAA GTT CGC |
| neg16 | CAC CGC AGC TCG TGT GTC GTA CTC |
| neg17 | CAC CGC GCC TTA AGA GTA CTC ATC |
| neg18 | CAC CGA GTG TCG TCG TTG CTC CTA |
| neg19 | CAC CGC AGC TCG ACC TCA AGC CGT |
| neg20 | CAC CGT ATC CTG ACC TAC GCG CTG |
| neg21 | CAC CGT GTA TCT CAG CAC GCT AAC |

**Supplementary Table 5. Antibodies used in these studies.**

| ***Western blotting*** |  |  |  |
| --- | --- | --- | --- |
| **Antigen** | **Clone** | **Manufacturer** | **ID #** |
| DYRK1A | D30C10 | Cell signaling | 8765 |
| MYC | D84C12 | Cell signaling | 5605S |
| pERK-Thr202/Tyr204 | D13.14.4E | Cell signaling | 4370S |
| ERK | 3A7 | Cell signaling | 9107S |
| BIM | C34C5 | Cell signaling | 2933S |
| BCL2 | 124 | Cell signaling | 15071S |
| CCND3 | DCS22 | Cell signaling | 2936S |
| Beta-Actin | AC-15 | Sigma-Aldrich | A5441 |
|  |  |  |  |
| ***Flow cytometry*** |  |  |  |
| **Antigen** | **Clone** | **Manufacturer** | **ID #** |
| CD19 | SJ25C1 | BD | 563325 |
| CD45 | 2D1 | Invitrogen | 17-9459-42 |

**Supplementary Table 6. RT-PCR primers used in these studies.**

| **Quantitative RT-PCR primers** | **Sequence** |
| --- | --- |
| DYRK1A | F_5-AGT TCT GGG TAT TCC ACC TGC TCA-3 |
|  | R_5-TGA AGT TTA CGG GTT CCT GGT GGT-3 |
| MYC | F_5-AGG GAG GTC CGG AGC GAA TA-3 |
|  | R_5-GTC CTT GCT CGG GTG TTG TA-3 |
| HOXA9 | F_5-CTG TCC CAC GCT TGA CAC TC-3 |
|  | R_5-CTC CGC CGC TCT CAT TCT C-3 |
| Meis1 | F_5-TCT GCA CTC GCA TCA GTA CC-3 |
|  | R_5-ATT GAC AGA GGA GCC CAT GC-3 |

**Supplementary Table 7. Targeted inhibitors used in these studies.**

| **Name** | **Vendor** | **ID #** |
| --- | --- | --- |
| GNF2133 | Moulder Center for Drug Discovery Research | NA |
| EHT1610 | Medchemexpress | HY-111380 |
| Venetoclax | Selleckchem | S8048 |
| Trametinib | Selleckchem | S2673 |

**Supplementary Table 8. DYRK1A sgRNA Sequences from CRISPR Kinome Screen**

| **Name** | **Sequence** | **Domain** |
| --- | --- | --- |
| DYRK1A_PKc_E4_75.3 | ATTGACTCCTTGATAGGCAA | Kinase domain |
| DYRK1A_PKc_E5_148 | TGTAAAGGCATATGATCGTG | Kinase domain |
| DYRK1A_PKc_E6_287.15 | GTTGCGCAAACTTTCGTGTT | Kinase domain |
| DYRK1A_PKc_E7_147.1 | TCGCTTTTATCGGTCTCCAG | Kinase domain |
| DYRK1A_PKc_E8_141.1 | GCTTGGTCAAGAATATGAGC | Kinase domain |
| DYRK1A_PKc_E9_213.21 | TTATGAAGTTTACGGGTTCC | Kinase domain |

**Supplementary References:**

1. Tarumoto Y, Lu B, Somerville TDD, Huang YH, Milazzo JP, Wu XS*, et al.* LKB1, Salt-Inducible Kinases, and MEF2C Are Linked Dependencies in Acute Myeloid Leukemia. *Mol Cell* 2018 Mar 15; **69**(6)**:** 1017-1027.e1016.

2. Tarumoto Y, Lu B, Somerville TDD, Huang Y-H, Milazzo JP, Wu XS*, et al.* LKB1, Salt-Inducible Kinases, and MEF2C Are Linked Dependencies in Acute Myeloid Leukemia. *Molecular Cell* 2018 2018/03/15/; **69**(6)**:** 1017-1027.e1016.

3. Kruth KA, Fang M, Shelton DN, Abu-Halawa O, Mahling R, Yang H*, et al.* Suppression of B-cell development genes is key to glucocorticoid efficacy in treatment of acute lymphoblastic leukemia. *Blood* 2017 Jun 1; **129**(22)**:** 3000-3008.

4. Tasian SK, Teachey DT, Li Y, Shen F, Harvey RC, Chen IM*, et al.* Potent efficacy of combined PI3K/mTOR and JAK or ABL inhibition in murine xenograft models of Ph-like acute lymphoblastic leukemia. *Blood* 2017 Jan 12; **129**(2)**:** 177-187.

5. Maude SL, Tasian SK, Vincent T, Hall JW, Sheen C, Roberts KG*, et al.* Targeting JAK1/2 and mTOR in murine xenograft models of Ph-like acute lymphoblastic leukemia. *Blood* 2012 Oct 25; **120**(17)**:** 3510-3518.

6. Loftus JP, Yahiaoui A, Shen F, Hurtz C, Brown PA, Tannheimer S*, et al.* Enhanced Efficacy of the SYK Inhibitor Entospletinib and Vincristine in KMT2A-Rearranged Acute Lymphoblastic Leukemia

*European Hematology Association Annual Congress* 2018**:** abstract PF164.

7. Hurtz C, Tasian SK, Wertheim GB, Ruggeri B, Stubbs MC, Perl AE*, et al.* Redundant JAK, SRC and PI3 Kinase Signaling Pathways Regulate Cell Survival in Human Ph-like ALL Cell Lines and Primary Cells. *Blood* 2017; **130**(Suppl 1)**:** 717-717.

8. Kempinska K, Malik B, Borkin D, Klossowski S, Shukla S, Miao H*, et al.* Pharmacologic Inhibition of the Menin-MLL Interaction Leads to Transcriptional Repression of PEG10 and Blocks Hepatocellular Carcinoma. *Mol Cancer Ther* 2018 Jan; **17**(1)**:** 26-38.

9. Wu Y, Doepner M, Hojnacki T, Feng Z, Katona BW, He X*, et al.* Disruption of the menin-MLL interaction triggers menin protein degradation via ubiquitin-proteasome pathway. *Am J Cancer Res* 2019; **9**(8)**:** 1682-1694.

10. Borkin D, He S, Miao H, Kempinska K, Pollock J, Chase J*, et al.* Pharmacologic inhibition of the Menin-MLL interaction blocks progression of MLL leukemia in vivo. *Cancer Cell* 2015 Apr 13; **27**(4)**:** 589-602.

11. Ianevski A, Giri AK, Aittokallio T. SynergyFinder 3.0: an interactive analysis and consensus interpretation of multi-drug synergies across multiple samples. *Nucleic Acids Res* 2022 May 17.

12. Ianevski A, Giri AK, Gautam P, Kononov A, Potdar S, Saarela J*, et al.* Prediction of drug combination effects with a minimal set of experiments. *Nature Machine Intelligence* 2019 2019/12/01; **1**(12)**:** 568-577.

13. Liu YA, Jin Q, Zou Y, Ding Q, Yan S, Wang Z*, et al.* Selective DYRK1A Inhibitor for the Treatment of Type 1 Diabetes: Discovery of 6-Azaindole Derivative GNF2133. *J Med Chem* 2020 Mar 26; **63**(6)**:** 2958-2973.

14. Dafflon C, Craig VJ, Méreau H, Gräsel J, Schacher Engstler B, Hoffman G*, et al.* Complementary activities of DOT1L and Menin inhibitors in MLL-rearranged leukemia. *Leukemia* 2017 Jun; **31**(6)**:** 1269-1277.

15. Krivtsov AV, Evans K, Gadrey JY, Eschle BK, Hatton C, Uckelmann HJ*, et al.* A Menin-MLL Inhibitor Induces Specific Chromatin Changes and Eradicates Disease in Models of MLL-Rearranged Leukemia. *Cancer Cell* 2019 Dec 9; **36**(6)**:** 660-673.e611.

16. Green MR, Monti S, Dalla-Favera R, Pasqualucci L, Walsh NC, Schmidt-Supprian M*, et al.* Signatures of murine B-cell development implicate Yy1 as a regulator of the germinal center-specific program. *Proc Natl Acad Sci U S A* 2011 Feb 15; **108**(7)**:** 2873-2878.

17. Hurtz C, Chan LN, Geng H, Ballabio E, Xiao G, Deb G*, et al.* Rationale for targeting BCL6 in MLL-rearranged acute lymphoblastic leukemia. *Genes Dev* 2019 Sep 1; **33**(17-18)**:** 1265-1279.

18. Duy C, Yu JJ, Nahar R, Swaminathan S, Kweon SM, Polo JM*, et al.* BCL6 is critical for the development of a diverse primary B cell repertoire. *J Exp Med* 2010 Jun 7; **207**(6)**:** 1209-1221.

19. Geng H, Hurtz C, Lenz KB, Chen Z, Baumjohann D, Thompson S*, et al.* Self-enforcing feedback activation between BCL6 and pre-B cell receptor signaling defines a distinct subtype of acute lymphoblastic leukemia. *Cancer Cell* 2015 Mar 9; **27**(3)**:** 409-425.

20. Swaminathan S, Huang C, Geng H, Chen Z, Harvey R, Kang H*, et al.* BACH2 mediates negative selection and p53-dependent tumor suppression at the pre-B cell receptor checkpoint. *Nat Med* 2013 Aug; **19**(8)**:** 1014-1022.

21. Liu YA, Jin Q, Zou Y, Ding Q, Yan S, Wang Z*, et al.* Selective DYRK1A Inhibitor for the Treatment of Type 1 Diabetes: Discovery of 6-Azaindole Derivative GNF2133. *Journal of Medicinal Chemistry* 2020 2020/03/26; **63**(6)**:** 2958-2973.

22. Loftus JP, Yahiaoui A, Brown PA, Niswander LM, Bagashev A, Wang M*, et al.* Combinatorial efficacy of entospletinib and chemotherapy in patient-derived xenograft models of infant acute lymphoblastic leukemia. *Haematologica* 2021 Apr 1; **106**(4)**:** 1067-1078.

23. Tasian SK, Hurtz C, Wertheim GB, Bailey NG, Lim MS, Harvey RC*, et al.* High incidence of Philadelphia chromosome-like acute lymphoblastic leukemia in older adults with B-ALL. *Leukemia* 2017 2017/04/01; **31**(4)**:** 981-984.

24. Hurtz C, Wertheim GB, Loftus JP, Blumenthal D, Lehman A, Li Y*, et al.* Oncogene-independent BCR-like signaling adaptation confers drug resistance in Ph-like ALL. *J Clin Invest* 2020 Jul 1; **130**(7)**:** 3637-3653.
